# Effects of pH, Temperature, and Light on the Inorganic Carbon Uptake Strategies in Early Life Stages of *Macrocystis pyrifera* (Ochrophyta, Laminariales)

**DOI:** 10.3390/plants13233267

**Published:** 2024-11-21

**Authors:** Bárbara S. Labbé, Pamela A. Fernández, July Z. Florez, Alejandro H. Buschmann

**Affiliations:** 1 Program of Magíster en Ciencias Mención Producción, Manejo y Conservación de Recursos Naturales, Universidad de Los Lagos, Puerto Montt 5400000, Chile; barbara.labbe@utas.edu.au; 2 Institute of Marine and Antarctic Studies (IMAS), University of Tasmania, Hobart, TAS 7005, Australia; 3 Centro i~mar, CeBiB & MASH, Universidad de Los Lagos, Puerto Montt 5400000, Chile; july.florez@upla.cl (J.Z.F.); abuschma@ulagos.cl (A.H.B.); 4 Departamento de Ciencias y Geografía, Facultad de Ciencias Naturales y Exactas, HUB Ambiental UPLA, Universidad de Playa Ancha, Valparaíso 2340000, Chile

**Keywords:** climate change, gametophytes, carbon uptake, kelp, ocean acidification, seaweed

## Abstract

The responses of seaweed species to increased CO_2_ and lowered pH (Ocean Acidification: OA) depend on their carbon concentrating mechanisms (CCMs) and inorganic carbon (Ci) preferences. However, few studies have described these mechanisms in the early life stages of seaweeds or assessed the effects of OA and its interactions with other environmental drivers on their functionality and photophysiology. Our study evaluated the effects of pH, light (PAR), temperature, and their interactions on the Ci uptake strategies and photophysiology in the early stages of *Macrocystis pyrifera*. Gametophytes were cultivated under varying pH (7.80 and 8.20), light (20 and 50 µmol photons m^−2^s^−1^), and temperature (12 and 16 °C) conditions for 25 days. We assessed photophysiological responses and CCMs (in particular, the extracellular dehydration of HCO_3_^−^ to CO_2_ mediated by the enzyme carbonic anhydrase (CA) and direct HCO_3_^−^ uptake via an anion exchange port). This study is the first to describe the Ci uptake strategies in gametophytes of *M. pyrifera*, demonstrating that their primary CCM is the extracellular conversion of HCO_3_^−^ to CO_2_ mediated by CA. Additionally, our results indicate that decreased pH can positively affect their photosynthetic efficiency and maximum quantum yield; however, this response is dependent on the light and temperature conditions.

## 1. Introduction

Anthropogenic greenhouse gas emissions are considered the primary driver of global change, causing increases in global sea surface temperatures (ocean warming: OW) and a global decrease in the pH of surface waters (ocean acidification: OA) [1]. Under the business-as-usual CO_2_ emissions scenario, sea surface temperatures are expected to increase globally by 2.0–4.3 °C, while the pH of surface waters may decline by 0.3 units by 2100 [1]. Decreasing surface water pH alters the carbonate chemistry of seawater (SW), causing changes in the concentrations of protons ([H^+^]) and the proportions of dissolved inorganic carbon (DIC) sources, including carbon dioxide (CO_2_), bicarbonate (HCO_3_^−^), and carbonate ions (CO_3_^−2^), each of which can differentially—as well as interactively—affect marine biota [2,3]. DIC and temperature are key factors influencing metabolic processes such as growth and photosynthesis in marine primary producers (i.e., phytoplankton and seaweeds); thus, the single and combined effects of OA and OW can substantially affect their physiological and ecological performance [4]. Along with OW and OA, light availability for benthic primary producers might also be reduced by anthropogenic activities (e.g., agriculture, forestry, pollution) [5,6], which may lead to decreased primary productivity in coastal ecosystems [7,8]. Thus, these global and local changes might directly affect the productivity rates, structure, and persistence of marine communities [9].

Seaweeds, including kelps (large brown algae of the Order Laminariales), have foundational functions in coastal ecosystems and are the base of the food web and major contributors to benthic primary production [9,10,11]. They provide food, habitat, and shelter to higher trophic levels (e.g., shellfish and fish) [2], as well as a range of ecosystem services, such as contributing to carbon and nitrogen cycling, climate regulation, and biodiversity [2,11,12]. In seaweed, as in all photosynthetic organisms, dissolved CO_2_ is the substrate for carbon fixation by the enzyme ribulose-1.5-bisphosphate carboxylase/oxygenase (RuBisCO) [2,13]. However, at the current SW pH (7.9–8.1), CO_2_ represents a small proportion of the total DIC and, although it diffuses passively into the cell, this process is too slow to saturate photosynthesis in most species [11]. Therefore, most seaweeds (~70%) have developed specialized carbon concentrating mechanisms (CCMs) to increase the CO_2_ concentration close to RuBisCO and thus reduce Ci limitations [13,14]. It is assumed that most CCMs are based on the direct or indirect use of HCO_3_^−^ from the bulk SW, representing about 91% of the total DIC [14]. These mechanisms include (1) HCO_3_^−^ extracellular dehydration to CO_2_, catalyzed by the external enzyme carbonic anhydrase (CA_ext_); (2) direct HCO_3_^−^ uptake through an anion exchange (AE) protein located at the plasmalemma; and (3) active uptake of CO_2_ or HCO_3_^−^, which involves a proton motive force through an H^+^-ATPase pump [15,16,17]. Therefore, the increased CO_2_ and HCO_3_^−^ concentrations associated with OA can strongly affect the photosynthetic physiology of seaweeds, depending on their CCMs and relative ability to use CO_2_ vs. HCO_3_^−^ [16,17,18,19]. However, while high-affinity CCMs might be down-regulated by increased dissolved CO_2_ in some cases [20,21], such conditions might not cause physiological alterations in non-CCM species [18,22]. Additionally, regardless of whether they have a CCM or not, the physiological responses of seaweeds to OA may also be influenced by other environmental drivers, such as temperature and light.

The combined effect of OA with temperature and light will undoubtedly impact the Ci acquisition strategies of seaweeds and, consequently, their photosynthetic and growth rates [11]. In the case of kelp species, these responses are even more complex due to their intricate life cycle, which alternates between microscopic (haploid: meiospores and gametophytes and diploid: embryonic sporophytes) and macroscopic (diploid: adult sporophyte) stages [9]. Across this complex life cycle, micro- and macroscopic life stages are exposed to contrasting environmental conditions in terms of light, temperature, and CO_2_/pH along the water column. These conditions can modulate ontogenetic differences in their tolerances and sensitivities to environmental changes [23,24,25]; for example, previous studies in the giant kelp *M. pyrifera* have shown that, at pH 7.86, meiospore germination is unaffected, while gametophyte size is either positively or negatively affected [26]. At pH 7.61, meiospore germination is reduced, but gametophyte size remains unchanged. This may be due to protective mechanisms that minimize the effects of H^+^ ions on seaweed cell metabolism, which could operate at the expense, for example, of growth [18,26]. Similar results have been described for other Laminariales species, such as *Undaria pinnatifida* (Harvey) [27]. Juvenile sporophytes and gametophytes of *U. pinnatifida* exhibit similar photosynthetic responses at pH of 7.3 and 8.3; however, when the pH is increased to 9.3, photosynthetic rates of gametophytes are reduced, while those in sporophytes remain unchanged [27]. Similarly, Hollarsmith et al. [28] observed distinct responses to temperature across the early life stages of Chilean *M. pyrifera* populations. Meiospores showed higher tolerance to elevated and low temperatures (18 °C and 12 °C, respectively), and gametophytes from all populations grew and survived under all temperature treatments (18 °C and 12 °C). However, gametophytes of all Chilean populations tested failed to reach sexual maturity and produce eggs at elevated temperatures (≥18 °C). These distinct responses to temperature across the life stages of Chilean *M. pyrifera* may be explained by the local environmental variability experienced throughout their life cycle.

Previous studies have predicted that the early life stages of kelp might be more susceptible to abrupt changes in temperature [25,29,30] and highly vulnerable to OA [31,32]. Therefore, it is fundamental to understand how these micro and macroscopic stages will respond to changing climatic scenarios regarding the physiology of kelps. A recent study on the kelp *Saccharina angustissima* (Laminariales, Phaeophyceae) showed that each stage of its life cycle exhibits different responses to changes in temperature and light [25]. For example, meiospore germination occurred at temperatures ranging from 7 to 17 °C under light conditions between 20 and 80 μmol photon m^−2^s^−1^, except at 12 and 15 °C, where 80 μmol photon m^−2^s^−1^ considerably reduced the success of germination. Gametophytes grew best at temperatures between 8 and 13 °C and at the lowest irradiance (10 μmol photon m^−2^s^−1^), with light availability having a positive effect only at high temperatures (15 and 17 °C). For juvenile sporophytes, temperatures between 8 and 15 °C and light intensities between 10 and 100 μmol photon m^−2^s^−1^ are suitable for growth, while higher temperatures and light intensities considerably reduce growth. These results suggest that the combination of OW and the effects of high light intensity could put pressure on the early life cycle stages of *S. angustissima*. These differences are likely due to the close relationship between the functionality of CCMs and light. Recent studies have suggested that the presence or expression of CCMs in seaweeds is strongly associated with light availability [33,34,35], due to the varying physiological energy costs of each mechanism [17,19,35]. Thus, in seaweeds, the energy saved through the expression or downregulation of these mechanisms can be allocated to other physiological processes, such as growth, photosynthesis, or reproduction. However, the effects of light availability on CCMs in the early stages of kelps are still unknown.

The giant kelp *M. pyrifera* forms extensive forests widely distributed along the north and south Pacific coast, inhabiting latitudinal gradients of temperature, nutrients, salinity, and upwelling zones [9,36]. *M. pyrifera* is a major contributor to coastal productivity [10,37] and plays a key role in marine ecosystems [38]. *M. pyrifera* presents a biphasic life cycle with alternate generations between micro and macroscopic stages [9]. The development of microscopic early stages of *M. pyrifera* involves complex processes that are not yet fully understood [39,40], which may be essential to explain the abundance and distribution patterns of *M. pyrifera* populations [41]. This study evaluated the independent and interactive effects of pH/CO_2_, light, and temperature on the carbon metabolism and photophysiology of *M. pyrifera* in its early life stages. The presence of CCMs was assessed using specific inhibitors: acetazolamide (AZ), which inhibits CA_ext_; etoxyzolamide (EZ), which inhibits both enzymes (CA_ext_ and CA_int_); and 4,4′-diisothiocyanatostilbene-2,2′-disulfonate (DIDS), which inhibits the direct uptake of HCO_3_^−^ via an AE protein [42]. *M. pyrifera* gametophytes were incubated under combined pH (pH 7.80 and 8.20), light PAR intensity (20 and 50 µmol photon m^−2^s^−1^), and temperature (12 °C and 16 °C) conditions for 25 days. At the end of the incubation, photosynthetic rates and photobiological aspects (i.e., Chlorophyll *a* fluorescence, and pigment concentrations) were measured in the gametophytes. We hypothesized that (1) the CCMs in the early stages of *M. pyrifera* differ from those described in adult individuals; and (2) the OA pH (7.80) positively affects the carbon metabolism and physiology of *M. pyrifera* gametophytes by increasing diffusive CO_2_ use, but this response is dependent on light availability and temperature. 

## 2. Results

### 2.1. Experiment 1: pH × Light

#### 2.1.1. The Effects of Specific Inhibitors on the Ci Acquisition Mechanisms

**Figure 1:**
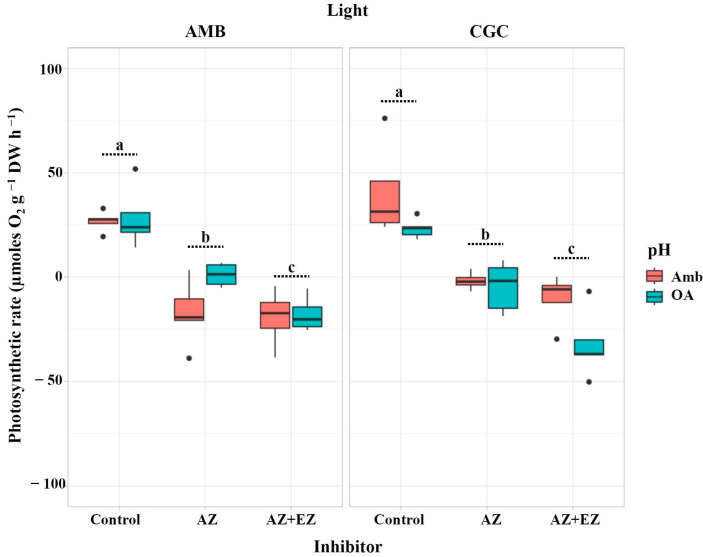
Photosynthetic rate (µmoles O_2_ g^−1^ DW h^−1^) in *M. pyrifera* gametophytes after injection of CA inhibitors from Experiment 1: pH × light. Color of legends indicates pH conditions: Ambient (Amb, red) and Ocean Acidification (OA, blue). Light condition panels: AMB Light (50 µmol photon m^−2^s^−1^) and CGC Light (20 µmol photon m^−2^s^−1^). Control: gametophytes without the inhibitors AZ or EZ. Values represent the mean ± SD (*p* < 0.005, three-way ANOVA). Different letters indicate significantly different values (*p* < 0.05).

After the injection of specific CA inhibitors (AZ alone and AZ + EZ), the photosynthetic rate of *M. pyrifera* gametophytes, expressed as µmol O_2_ g^−1^ DW h^−1^, was significantly reduced across all treatment combinations, reaching values close to zero and even turning negative (Figure 1). Our results showed that, regardless of the pH and light conditions, the photosynthetic rate was completely inhibited by the presence of the inhibitors (*p* = 5.64 × 10^−14^; Table 1). The inhibitory effect on the photosynthetic rate was significantly affected by the pH × light interaction (*p* = 0.0048; Table 1), but not by pH (*p* = 0.3706) or light (*p* = 0.5369, Table 1) individually.

**Figure 2:**
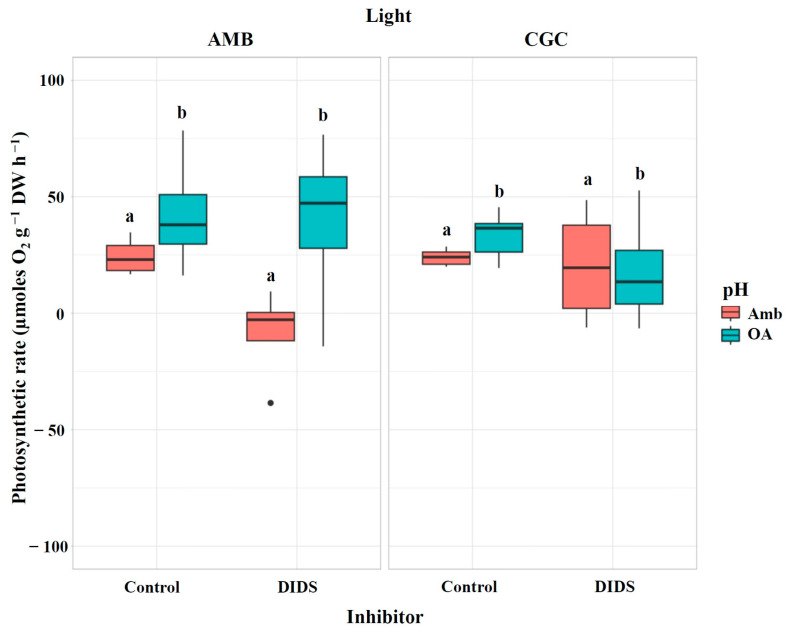
Photosynthetic rate (µmoles O_2_ g^−1^ DW h^−1^) in *M. pyrifera* gametophytes after injection of the direct HCO_3_^−^ uptake inhibitor from Experiment 1: pH × light. Color of legends indicates pH conditions: Ambient (Amb, red) and Ocean Acidification (OA, blue). Light condition panels: AMB Light (50 µmol photon m^−2^s^−1^) and CGC Light (20 µmol photon m^−2^s^−1^). Control: gametophytes without the inhibitor DIDS. Values represent the mean ± SD (*p* < 0.005, three-way ANOVA). Different letters indicate significantly different values (*p* < 0.05).

Following the injection of the direct HCO_3_^−^ uptake inhibitor (DIDS), the photosynthetic rates of *M. pyrifera* gametophytes remained unchanged (*p* = 0.0748; Table 1; Figure 2). A slight decrease in the photosynthetic rate was observed under ambient pH and light conditions, compared to low light (Climate Global Change: CGC scenario), but this difference was not statistically significant (*p* = 0.0574; Table 1). Additionally, there were no significant interactions between pH, light, and the inhibitor (*p* = 0.9702; Table 1).

#### 2.1.2. Photosynthetic Pigment Analysis

The Chl *c/*Chl *a* ratio in *M. pyrifera* gametophytes was significantly affected by light intensity (*p* = 0.0003, Table 1), but not by pH or its interaction with light (*p* = 0.0951; *p* = 0.7062, Table 1). The Chl *c/*Chl *a* ratio was higher at 50 µmol photon m^−2^s^−1^, compared to 20 µmol photon m^−2^s^−1^, regardless of pH treatment (Ambient pH: 8.20 and OA pH: 7.80; Table 2). The Fucox/Chl *a* ratio was significantly affected by light and pH, but not by their interaction (*p* = 0.4303; Table 1). The ratio was higher at 50 µmol photon m^−2^ s^−1^, compared to 20 µmol photon m^−2^s^−1^ (Table 2). Under ambient light conditions, the Fucox/Chl *a* ratio was considerably higher at pH 7.80, reaching values of 1.52 at 50 µmol photon m^−2^s^−1^; however, these differences were not observed under CGC light conditions at pH 8.20 (Table 2). Our results indicated a higher concentration of Chl *a* and proportion of photosynthetic pigments under lower light conditions, with a greater concentration of Chl *a* observed at 20 µmol photon m^−2^s^−1^ compared to 50 µmol photon m^−2^s^−1^ (*p* = 0.001, Table 1).

#### 2.1.3. Photophysiology: Chlorophyll *a* Fluorescence

The photosynthetic efficiency (α) of *M. pyrifera* gametophytes was significantly affected by pH, but not by light or its interaction with pH (*p* = 0.6253, *p* = 0.7555; Table 1). At pH 7.80, α was significantly higher than that at pH 8.20, with values of 0.31 and 0.16 µmol photon m^−2^s^−1^, respectively (Table 1).

**Figure 3:**
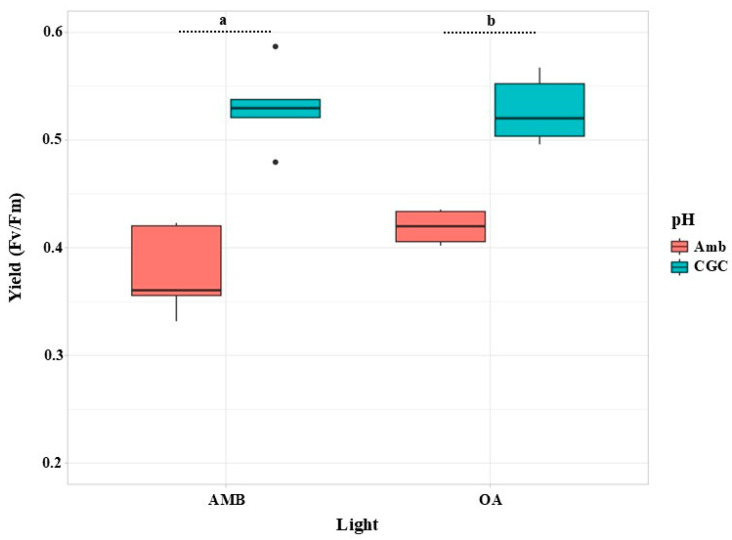
Maximum quantum yield (F_v_/F_m_) in *M. pyrifera* gametophytes in Experiment 1 (corresponding to pH × light). pH conditions: Ambient (AMB) and Ocean Acidification (OA). Color of legends indicates temperature conditions: Ambient (Amb, 12 °C) and Climatic Global Changes (CGC, 16 °C). Values represent the mean ± SD (*p* < 0.005, two-way ANOVA). Different letters indicate significantly different values (*p* < 0.05).

The maximum quantum yield (F_v_/F_m_) of *M. pyrifera* gametophytes was significantly affected by pH, but not by light or its interaction with pH (*p* = 0.275; *p* = 0.181; Table 1). At pH 7.80, F_v_/F_m_ was higher compared to that at pH 8.20, with values of 0.53 and 0.44, respectively (Table 1; Figure 3). This suggests that, under future OA conditions (pH 7.80), F_v_/F_m_—which is known to be a parameter indicative of physiological stress—would not be negatively impacted in *M. pyrifera* gametophytes.

The ETR_max_ of *M. pyrifera* gametophytes was significantly affected by light and the interaction of pH × light, but not by pH alone (*p* = 0.0001; *p* = 0.0072; *p* = 0.3216; Table 1). At both pH 7.80 and pH 8.20, ETR_max_ was higher at 20 µmol photon m^−2^s^−1^ when compared to 50 µmol photon m^−2^s^−1^; notably, this difference was greater at ambient pH than at lowered pH (Table 1 and Table 3). Similarly, E_k_ values were significantly affected by pH, light, and their interaction (*p* = 0.0231; *p* = 0.0083; *p* = 0.0191; Table 1). At both pH 7.80 and pH 8.20, E_k_ values were higher at 20 µmol photon m^−2^s^−1^ compared to 50 µmol photon m^−2^s^−1^; however, this difference was greater at ambient pH than at lowered pH (Table 1 and Table 3).

### 2.2. Experiment 2: pH × Temperature

#### 2.2.1. The Effects of Specific Inhibitors on the Ci Acquisition Mechanism

**Figure 4:**
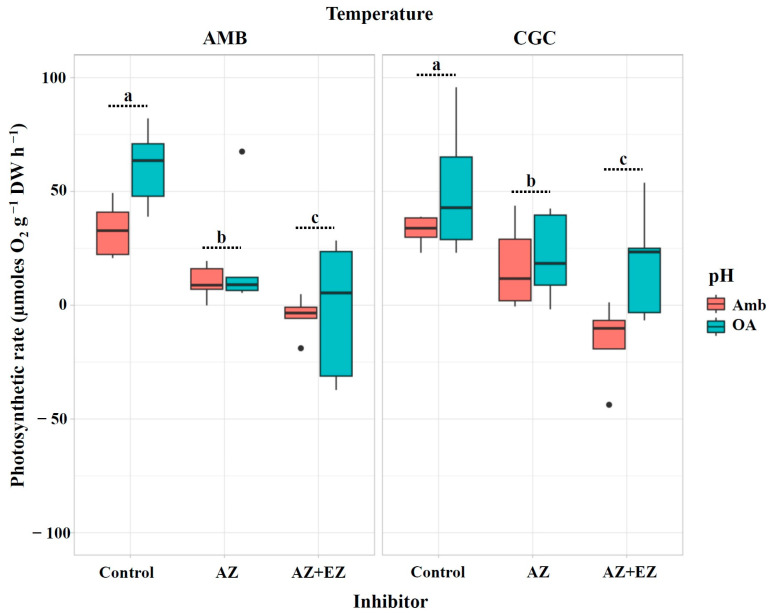
Photosynthetic rate (µmoles O_2_ g^−1^ DW h^−1^) in *M. pyrifera* gametophytes after injection of CA inhibitors in Experiment 2: pH × temperature. Color of legends indicates pH conditions: Ambient (Amb, red) and Ocean Acidification (OA, blue). Temperature condition panels: AMB (12 °C) and CGC (16 °C). Control: gametophytes without inhibitors (AZ and EZ). Values represent the mean ± SD (*p* < 0.005, three-way ANOVA). Different letters indicate significantly different values (*p* < 0.05).

After the injection of AZ—a specific CA_ext_ inhibitor—the photosynthetic rate of *M. pyrifera* gametophytes was considerably reduced across all treatment combinations, reaching values close to zero and even negative values (Figure 4). The inhibitory effect was more pronounced under ambient pH conditions, compared to the OA treatment (*p* = 0.0024), regardless of the temperature treatment (*p* = 0.5086; Table 4; Figure 4).

After the simultaneous injection of both CA inhibitors (i.e., AZ and EZ), the photosynthetic rate of *M. pyrifera* gametophytes did not experience further inhibition in any pH treatment, compared to the photosynthetic rate after the injection of AZ alone, regardless of the temperature (Figure 4).

**Figure 5:**
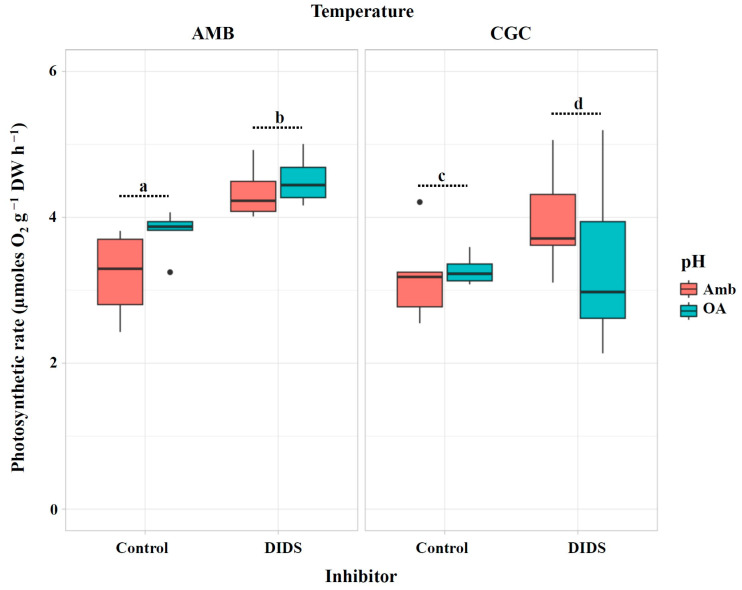
Photosynthetic rate (µmoles O_2_ g^−1^ DW h^−1^) in *M. pyrifera* gametophytes after injection of the direct HCO_3_^−^ uptake inhibitor in Experiment 2: pH × temperature. Color of legends indicates pH conditions: Ambient (Amb, red) and Ocean Acidification (OA, blue). Temperature condition panels: AMB (12 °C) and CGC (16 °C). Control: gametophytes without inhibitor (DIDS). Values represent the mean ± SD (*p* < 0.005, three-way ANOVA). Different letters indicate significantly different values (*p* < 0.05).

Following injection of the direct HCO_3_^−^ uptake inhibitor (DIDS), the photosynthetic rate of *M. pyrifera* gametophytes increased slightly across all pH × temperature treatments (Figure 5). This increase was more pronounced under CGC temperature (*p* = 0.027; Table 4); however, this effect may be attributed to an experimental issue, such as DIDS dissolution, and it is not considered physiologically relevant for photosynthesis.

#### 2.2.2. Photosynthetic Pigment Analysis

The Chl *c*/Chl *a* ratio of gametophytes of *M. pyrifera* was significantly affected by the pH × temperature interaction (*p* = 0.0449; Table 4). At the ambient temperature treatment, the Chl *c*/Chl *a* ratio showed greater values at pH 7.80 than at pH 8.20, reaching values of 0.88 and 0.69, respectively (Table 1). However, this increase in the Chl *c*/Chl *a* ratio was not observed under CGC temperature conditions. The Fucox/Chl *a* ratio of gametophytes of *M. pyrifera* was significantly affected by temperature, but not by pH or pH × temperature (*p* = 0.0032; *p* = 0.4655; Table 2), showing greater values at 16 °C than 12 °C (0.84 and 0.88, respectively), regardless of pH treatment (Table 4).

#### 2.2.3. Photophysiology: Chlorophyll *a* Fluorescence

The photosynthetic efficiency (α) of *M. pyrifera* gametophytes was significantly affected by the interaction between pH and temperature (*p* = 1.415 × 10^−06^; Table 4). At pH 8.20, α was significantly higher at 12 °C, compared to all other treatments (Table 2).

**Figure 6:**
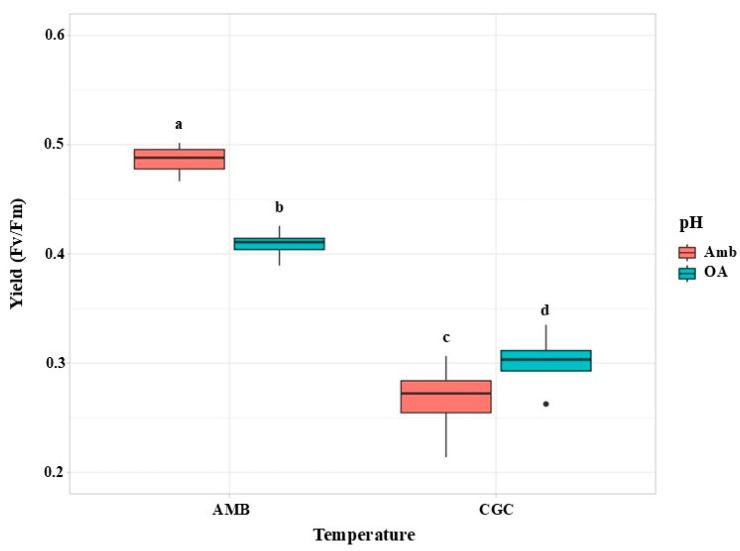
Maximum quantum yield (F_v_/F_m_) in *M. pyrifera* gametophytes from Experiment 2 corresponding to pH × temperature. pH conditions: Ambient (AMB) and Ocean Acidification (OA). Color of legends indicates temperature conditions: Ambient (Amb, 12 °C) and Climatic Global Changes (CGC, 16 °C). Values represent the mean ± SD (*p* < 0.005, two-way ANOVA). Different letters indicate significantly different values (*p* < 0.05).

The maximum quantum yield (F_v_/F_m_) of *M. pyrifera* gametophytes was significantly affected by the interaction between pH and temperature (*p* = 0.0003; Table 4). F_v_/F_m_ was considerably reduced under CGC temperature scenarios (16 °C), when compared to ambient temperature (12 °C) under both pH treatments (Figure 6). At pH 8.2, F_v_/F_m_ was significantly higher at 12 °C than at 16 °C, with values of 0.49 and 0.29, respectively (Figure 6). Furthermore, F_v_/F_m_ at pH 8.20 and 12 °C was significantly higher, when compared to all temperature treatments at pH 7.80 (Table 3 and Table 4; Figure 6).

The ETR_max_ of *M. pyrifera* gametophytes was significantly affected by pH and temperature, but not by their interaction (*p* = 0.0262; *p* = 0.0193; *p* = 0.058; Table 4). ETR_max_ was higher at ambient pH than at lowered pH, reaching the highest value at 12 °C (Table 3). Contrary to ETR_max_, E_k_ values were not significantly affected either by pH, light, or their interaction (*p* = 0.2139; *p* = 0.8207; *p* = 0.5629; Table 3 and Table 4).

## 3. Discussion

Our results support the hypothesis that CCMs in the early life stages of *M. pyrifera* differ from those described in adult individuals. We found that the photosynthetic rates of *M. pyrifera* gametophytes under all pH (7.80 and 8.20), light (20 and 50 µmol m^−^^2^s^−^^1^), and temperature (12 and 16 °C) treatments were 80–100% inhibited by the specific CA inhibitors (AZ + EZ). To the contrary, in the presence of the external HCO_3_^−^ direct uptake inhibitor (DIDS), their photosynthetic rates mainly remained unaffected. These findings confirm that *M. pyrifera* gametophytes are able to use HCO_3_^−^ from the external medium to support their photosynthesis via a CA_ext_-mediated pathway, with the AE-type transmembrane transporter having a small contribution. These results are contrary to what has been described in adult individuals of *M. pyrifera*, where the use of HCO_3_^−^ is primarily dependent on the direct HCO_3_^−^ uptake via an AE port, with CA_ext_ having little contribution [17]. However, the photosynthetic rates of gametophytes under ambient pH (8.20) and light (50 µmol m^−^^2^s^−^^1^) conditions were partially inhibited by the presence of the direct HCO_3_^−^ uptake inhibitor, suggesting that this mechanism might support photosynthesis in *M. pyrifera* gametophytes under certain environmental conditions.

The external dehydration of HCO_3_^−^ to CO_2_ by the enzyme CA_ext_ enhances the CO_2_ concentration at the cell surface, facilitating the diffusion of CO_2_ into the cell [43,44]. This mechanism can be less energetically expensive than direct HCO_3_-uptake via the AE port and other CCMs such as direct CO_2_ uptake, implying that the energy saved might be directed to support other physiological processes such as growth [45]. Our results support the hypothesis previously raised by Leal et al. [32] who, when studying the effects of OA and elevated temperature on the early stages of this species, suggested that they may use CO_2_ either through passive diffusion or via the CA_ext_, due to the enhanced growth rates observed at lowered pH (7.20). Similarly, the highest growth rates of *M. pyrifera* gametophytes were observed under lowered pH (7.86 and 7.61) by Roleda et al. [26], suggesting that increased CO_2_ or greater [H^+^] concentrations might favor the growth of *M pyrifera* in early life stages. The presence of CA enzymes has been successfully detected in other early life stages of large brown seaweeds; for example, both CA_ext_ and CA_int_ have been characterized in gametophytes of *S*. *japonica* using molecular and cellular methods [46,47,48,49], with no HCO_3_^−^ transporters detected [50]. Those results suggest that gametophytes of *S*. *japonica* might be unable to directly take up HCO_3_^−^ from the external medium. Similarly, Zhang et al. [27] showed that gametophytes and sporophytes of *U. pinnatifida* use HCO_3_^−^ via the CA_ext_-mediated pathway to support their photosynthesis, with gametophytes having a lower affinity for HCO_3_^−^ than juvenile sporophytes. In red seaweed species, which exhibit a different life cycle than kelps, it has also been demonstrated that Ci utilization varies between gametophytes and sporophytes. For example, Wang et al. [50] showed that, while the thallus (haploid) of *Pyropia haitanensi* mainly depends on CA_ext_ to acquire Ci, the conchocelis (diploid) uses both HCO_3_^−^ utilization mechanisms; that is, AE transporters and CA_ext_ and CA_int_. These findings and our results strongly suggest that Ci uptake strategies can vary among the life stages (haploid vs. diploid) of seaweed species. These differences might be due to the differential energetic requirements across its life cycle; for example, early life stages might utilize less energetically costly Ci uptake strategies to support other physiological processes, such as growth and/or reproduction, or due to local environmental conditions.

In seaweeds, the existence or expression of different HCO_3_^−^ utilization mechanisms can be influenced by external environmental conditions [51]; for example, species with moderate to high CCM capacity typically thrive in high-light environments with sufficient energy to support energetically expensive CCMs. Conversely, higher reliance on diffusive CO_2_ is commonly found in low-light environments [13,50,52]. These differences have even been observed within the same species; for example, in *Ulva* sp. growing in cold waters with low irradiance, the CA-mediated pathway predominates. In contrast, direct HCO_3_^−^ uptake via the AE port predominates in *Ulva* sp. growing in warmer waters with higher irradiance [53]. The latter mechanism of HCO_3_^−^ utilization is more efficient than the CA-mediated pathway. Hence, it can support high photosynthetic rates more efficiently, which typically occur under conditions of high temperature and high irradiance [53]. This advantage of alternating between Ci mechanisms could also occur in kelp stands, considering their global distribution and complex life cycles, which expose them to a wide range of temperatures, nutrient availability, hydrodynamic stresses, and light conditions [9,36].

Kelps inhabit subtidal habitats down to depths of 20–30 m, with settlement stages (gametophytes, embryonic sporophytes) growing at the upper limit zone, typically under the shade of the adult canopy with little exposure to high irradiances [9,54,55]. Thus, similar to the light-limited deep blades of *M. pyrifera*, which are well adapted to shade and exhibit lower photosynthetic rates than canopy blades [50,56,57], gametophytes and embryonic sporophytes also exhibit lower light requirements to support photosynthesis, when compared to the adult sporophytes (see [56] and the references therein). This could explain the predominance of the CA-mediated pathway for utilizing HCO_3_^−^. Given the environmental conditions to which these life stages are exposed—namely, low light and cold waters—the CA-mediated pathway is likely sufficient to supply CO_2_ to RuBisCO and support photosynthesis. Our findings, along with previous studies on the species [10,16,31], confirm its capacity to alternate its mode of HCO_3_^−^ utilization depending on its environmental conditions and physiological requirements.

We also hypothesized that, under lowered pH (7.80), the carbon metabolism and photophysiology of *M. pyrifera* gametophytes will be positively affected by increased CO_2_ use; however, this response would depend on the availability of light and temperature. Contrary to our hypothesis, we did not observe a significant effect of lowered pH on the functioning of CCMs under any of the light or temperature treatments. For example, the impact of specific CA inhibitors on the photosynthetic rates of gametophytes was similar at both ambient and lowered pH, suggesting that CA activity was not downregulated by increased CO_2_ availability (i.e., enhanced CO_2_ use via passive diffusion). However, other physiological parameters showed differential responses to lowered pH and its interaction with light and temperature. We found that the gametophytes’ photosynthetic efficiency (α) and maximal quantum yield (F_v_/F_m_) were higher at lowered pH than at ambient pH, regardless of light availability. These results suggest that OA (lowered pH and increased CO_2_ availability) may increase photosynthesis in gametophytes of *M. pyrifera*. However, further metabolic studies are needed to elucidate the potential benefits associated with lowered pH and increased CO_2_ in the early life stages of kelps.

Conversely, our results indicated that a future decrease in light intensity is unlikely to impact the photosynthetic efficiency of *M. pyrifera* gametophytes negatively. This gametophytic resiliency may be attributed to their high acclimatization capacity to varying light intensities [58,59] and their relatively low light requirements to support photosynthesis (see [56] and the references therein). The low light treatment (20 µmol m^−^^2^ s^−^^1^) applied throughout our study may not have limited their photosynthesis. We found that Chl *a* concentrations were higher under the low light treatment, compared to 50 µmol m^−^^2^ s^−^^1^ treatment, reaching values up to 0.12 mg g^−^^1^ WW. Despite the lack of studies describing the pigment content in this life cycle stage of *Macrocystis*, a previous study in *L. digitata* gametophytes described similar values to our study, ranging from 0.042 to 0.139 mg g^−^^1^ WW [60]. Additionally, it has been shown that gametophytes can ameliorate the negative effects of photodamage through the upregulation of accessory pigments [60], which supports our findings. We observed that the Chl*c*/Chl*a* and fucox/Chl*a* ratios varied significantly between light treatments (20 and 50 µmol m^−^^2^ s^−^^1^). In particular, these ratios were higher under 50 µmol m^−^^2^ s^−^^1^ compared to 20 µmol m^−^^2^ s^−^^1^, suggesting that gametophytes were able to upregulate their accessory pigments while downregulating Chl *a* concentrations. This is a clear indicator of photoacclimation in algal species [58,61]. An increase in the concentrations of secondary pigments could enhance their ability to capture light and, thus, provide the energy to generate ATP and NADPH under conditions of greater CO_2_ availability [53]. Classical studies on *M. pyrifera* have shown how the composition of the pigments Chl *a*, Chl *c*, and fucoxanthin vary in juvenile sporophytes of *M. pyrifera* after they were transplanted at different depths [62]. These processes of variation and acclimatization have also been reported in other brown algae, such as *Sargassum* [63] or *Ascoplhyllum nodosum* [64]. A previous study on gametophytes of *L. digitata* showed that these stages exhibit great plasticity in their photosynthetic responses to light and temperature conditions [60]. Such physiological plasticity may enable gametophytes to thrive in diverse environments; this trait can be important for maintaining *M. pyrifera* populations in future oceans.

We also observed an interactive effect of OA and temperature on photosynthetic efficiency (α). Under OA pH conditions, α was similar under both temperature treatments. In contrast, at ambient pH, α was more than twice as high at 12 °C, when compared to the CGC temperature (16 °C). Similar to our study, Debelecq et al. [60] showed that the α value in gametophytes of *L. digitata* decreases with increasing temperature. However, increases in temperature also resulted in increases in the ETR_max_ and E_k_ values, which disagree with our results. The differences observed in our study regarding α values among temperature treatments were correlated with pigment content (Chl *a* and fucoxanthin), which decreased under the CGC temperature at ambient pH. This reduction in antennae pigments likely diminished light absorption at the higher temperature, contributing to a decrease in α [65]. Similarly, other photosynthetic parameters, such as F_v_/F_m_ and ETR_max_, were also reduced under elevated temperature at ambient pH. Interestingly, the negative impacts of elevated temperature on *M. pyrifera* gametophytes were observed only at ambient pH, and not under lowered pH. It has been shown that lower concentrations of pigments may limit energy transfer in the PS II [50], which could explain the low F_v_/F_m_ values observed in gametophytes grown at 16 °C under ambient pH. Similar to those in our study, the F_v_/F_m_ values in gametophytes of *L. digitata* decreased with increasing temperature. At the optimal temperature of 10 °C, F_v_/F_m_ values ranged from 0.52 to 0.53 in gametophytes of *L. digitata*, showing a progressive decline with increasing temperature. In our study, the F_v_/F_m_ values ranged from 0.29 at 16 °C to 0.49 at 12 °C, agreeing with the values described for *L. digitata* under optimal temperature conditions. However, additional information such as the tolerance and functionality of PSII—which is essential for the whole photochemistry process [60]—in the early life stages of kelps is still lacking.

## 4. Materials and Methods

### 4.1. Study Area and Sporophylls Collection

**Figure 7:**
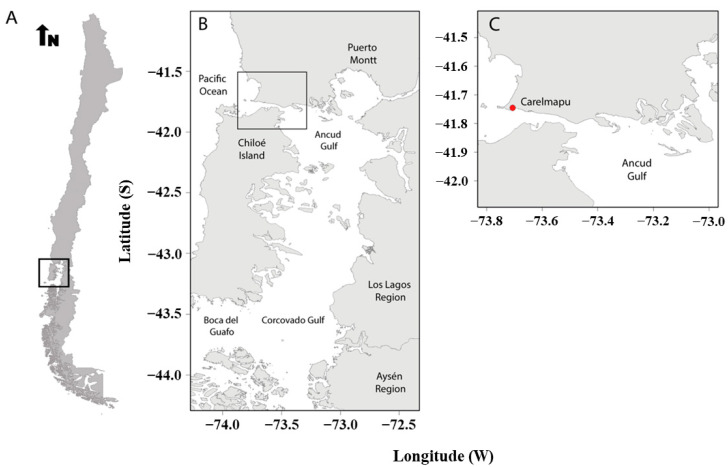
Sampling site of *Macrocystis pyrifera* sporophylls. (**A**) Map of Chile showing the study area. (**B**) Map of Southern Los Lagos Region with a zoom to Carelmapu locality (box). (**C**) Map of Carelmapu that indicates the specific study area (red point).

Fertile sporophylls were collected from ten sporophytes of *M. pyrifera* during low tide from the open coast site Carelmapu (Figure 7), Region de Los Lagos, South of Chile (41°44′45′′ S, 73°42′23′′ O) in February 2019. Sporophylls were transported to the laboratory using insulated containers filled with local seawater (SW). At the laboratory, eight to ten sporophylls (with mature sori) were dissected using a scalpel, and small disks (8–10 cm) were punched out from each sporophyll. These disks were gently rinsed, and any visible epibionts were brushed off with filtered ambient SW (0.22 μm pore, Merck Millipore, Burlington, MA, USA, at 12 °C). Later, the sporophyll disks were stored (wrapped in tissue and foil paper to induce dehydration) overnight at 4 °C before inducing meiospore release [66].

### 4.2. Experimental Design

Two independent experiments were conducted and, in each experiment, at least 5 to 10 disks with mature sori were immersed for 20 min in the respective pH_T_ treatments: 7.80 and 8.20 (pH measured on the total scale) to obtain a stock meiospore suspension. An initial concentration of 25,000 cells mL^−1^ was separately inoculated into 6 small Petri dishes containing 20 mL SW with the corresponding pH_T_ treatment (n = 6 independent replicates per pH_T_ treatment). The initial meiospore density was calculated using a 0.1 mm depth hemocytometer (Neubauer improved bright-line, Marienfeld, Germany). Meiospores were allowed to settle for 12 h, and the culture medium was then renewed to eliminate unsettled meiospores and detritus.

#### 4.2.1. Experiment 1: pH × Light

Meiospores of *M. pyrifera* were cultivated for 25 days in two identical, light-controlled growth chambers. One growth chamber was set up at 40 ± 5 µmol photon m^−^^2^s^−^^1^, representing the current light intensities (Ambient Light) described for the study site at a depth of 2 m. The other growth chamber was set up at 15 ± 5 µmol photon m^−^^2^s^−^^1^, simulating reduced light availability (Low Light), which can occur under the canopy in coastal marine ecosystems. Each growth chamber had a photoperiod of 16/8 h L:D (Light/Dark) PAR with a temperature of 12 °C, corresponding to the conditions at the study site. The light was provided using LED lamps (Philips, LED-EM-HO) and monitored with a PAR sensor (LI-COR, meter LI-250A, LI-COR, Lincon, NE, USA). For each light treatment, meiospore cultures were prepared at 25,000 cells mL^−1^ and the SW pH was adjusted to pH 7.80 (n = 6) or 8.20 (n = 6), simulating projected and current pH scenarios, respectively (see below for details of SW manipulation). Control cultures (SW without meiospores) corresponding to each pH × light treatment were prepared, and pH was monitored throughout the experiment. The culture medium was renewed every 3 days during the first 15 days of the experiment, and then every 1–2 days to prevent nutrient depletion. The pH of the culture medium was measured every 3–4 days, both after and before each medium renewal.

#### 4.2.2. Experiment 2: pH × Temperature

Meiospores of *M. pyrifera* were cultivated for 25 days in two identical, temperature-controlled rooms. One room was set up at 12 °C, representing the current temperature (Ambient Temp) at the study site, and the other was set up at 16 °C (12 °C + ~4 °C), simulating future OW conditions [1]. Each controlled room had a photoperiod of 16/8 h L:D (Light/Dark) with a light intensity of 40 ± 5 µmol photon m^−^^2^s^−^^1^, representing the current light intensity described for the study site (Ambient Light) at a depth of 2 m. The light was provided using LED lamps (Philips, LED-EM-HO) and monitored with a PAR sensor (LI-COR, meter LI-250A, LI-COR, Lincon, NE, USA). For each temperature treatment, meiospore cultures were prepared at 25,000 cells mL^−1^, and the SW pH was adjusted either to pH 7.80 (n = 6) or 8.20 (n = 6), representing the OA and AMB pH scenarios, respectively (see below for details of SW manipulation). Control cultures (SW without meiospores) corresponding to each pH × temperature treatment were prepared, and their pH was monitored throughout the experiment. The culture medium was renewed every 3 days during the first 15 days of the experiment, and then every 1–2 days to prevent nutrient depletion. The pH of the culture medium was measured every 3–4 days, both after and before each medium renewal.

At the end of both experiments, we evaluated the effects of specific inhibitors—AZ, which inhibits CA_ext_; EZ, which inhibits both enzymes (CA_ext_ and CA_int_); and DIDS, which inhibits the direct uptake of HCO_3_^−^ via an AE protein—on the photosynthetic rates of *M. pyrifera* gametophytes. We also measured the concentrations of photosynthetic pigments (Chl *a*, Chl *c*, and Fucoxanthin) and Chlorophyll *a* fluorescence, as described below (see Section 4.5).

### 4.3. Seawater pH Manipulations

At the laboratory, the onsite SW was filtered at 0.2 µm using a vacuum filter system and stored overnight in sterilized 2 L Schott Duran^®^ bottles at the respective experimental temperatures. After filtration and nutrient enrichment with Provasoli culture media [67], the ambient SW pH was 8.13. The two SW pH_T_ (pH measured on the total scale) treatments, 7.80 and 8.20, were prepared daily at the respective pH and temperature before renewing the culture medium. The different SW pH_T_ treatments were achieved using the acid/base method, mimicking the changes in SW carbonate chemistry due to OA [68]. To achieve the OA pH (7.80), equal volumes of 0.5 M HCl and 0.5 M NaHCO_3_ were used; meanwhile, for the SW ambient pH (8.20), equal volumes of 0.5 M NaOH and 0.5 M NaHCO_3_ were used.

### 4.4. Seawater pH Measurements

Seawater pH_T_ was measured at 12 °C using a pH electrode (Thermo Scientific Orion ROSS Sure-Flow semi-micro, ORI8175BNWPW, Massachusetts, US) connected to a pH meter (Thermo Scientific Orion 720A pH/ION Meter, Walthma, MA, USA). Temperature-equilibrated pH buffers (pH 7.0 and pH 10.0, color-coded, NIST traceable, Thermo Scientific) were used to calibrate the electrode and determine its slope. A TRIS buffer, standardized against a seawater buffer, was then used to measure pH on the total scale [69]. Seawater samples representing both treatments—7.80 (OA scenario) and 8.20 (AMB scenario)—were collected and fixed with mercuric chloride for subsequent carbonate chemistry analysis. The total alkalinity (A_T_) was measured according to the potentiometric method with a controlled titration closed cell (Metrohm, 848 Titrino Plus, Herisau, Switzerland) [69]. The SW carbonate chemistry of each pH treatment was calculated from the measured A_T_, pH_T_, salinity, and temperature (Table 1) using the SWCO2 software (version 2.3) [70].

### 4.5. Physiological and Biochemical Parameters

#### 4.5.1. Specific Inhibitors of Ci Acquisition Mechanism

We evaluated the Ci acquisition mechanisms of *M. pyrifera* gametophytes at the end of each experiment using specific inhibitors. The highly specific inhibitors used were AZ, which only inhibits CA_ext_, as it is poorly permeable with respect to passage through the plasma membrane [42,71]; EZ, which inhibits both enzymes (CA_ext_ and CA_int_), as it passes quickly and easily across the cell membrane [17,72]; and DIDS, which inhibits the direct uptake of HCO_3_^−^ via an AE port [17,73]. These inhibitors have been widely used in algae to study photosynthetic Ci acquisition mechanisms (e.g., [42,73]).

AZ and EZ were prepared in 0.02 M NaOH solutions [42], while DIDS was dissolved in MiliQ water. The final concentrations of the injected inhibitors were 100 µM for AZ and EZ, and 300 µM for DIDS. Each inhibitor was applied separately. The relative contribution of each Ci acquisition mechanism was estimated according to the effect of each inhibitor on the photosynthesis of the *M. pyrifera* gametophytes. Approximately 0.02 g of gametophytes from each Petri dish was transferred separately to Eppendorf tubes (1.5 mL) containing culture medium corresponding to each pH treatment. The tubes were incubated for 12 h before measurement, in order to minimize physiological stress. For each Petri dish (n = 5), O_2_ evolution was recorded using a fiber optic oxygen meter (Microx TX3, PreSens, Ragensburg, Germany, with temperature compensation and needle-type oxygen microsensors; PreSens, Ragensburg, Germany) for 20 min (initial photosynthesis time) under the light and temperature conditions of each treatment. After this period, the inhibitors were injected separately. For CA inhibitors, the order of injection was AZ first, followed by EZ. After the injection of each inhibitor, O_2_ evolution was measured for an additional 10 min (total measurement time per sample: 20 min initial + 10 min AZ + 10 min EZ). For DIDS, O_2_ evolution was measured continuously for 10 min post-injection. O_2_ evolution was also measured for control treatments for pH, as described above. The effect of each inhibitor was calculated through a linear regression during the last 5 min of incubation. The measurements were standardized according to the dry weight of the corresponding sample (g) and expressed as μmol O_2_ g^−^^1^ dried algae h^−^^1^ (modified from [42]).

#### 4.5.2. Chlorophyll *a* Fluorescence

Chlorophyll *a* fluorescence of photosystem II (PSII) was measured on the last day of the experiment using a pulse amplitude modulated fluorometer Junior-PAM (Heinz Walz, Effeltrich, Germany), provided with an Emitter–Detector Unit (ED). Gametophytes were carefully detached from each Petri dish by hand, sieved through a 20 µm mesh, and then placed in a 96-well microplate (400 µL) (Costar-96-well model) with culture medium corresponding to each pH_T_ treatment. Measurements were performed in each of the 5 experimental units (microplate well) by positioning the optical fiber (1.5 mm) directly inside each experimental unit, maintaining a constant distance of 10–15 mm between the optical fiber and the bottom of the microplate well. The potential maximum quantum yield (F_v_/F_m_) was determined according to Hanelt [74]. After a 15 min dark adaptation period, the minimal F_0_ was recorded with a pulsed measuring light of 650 nm followed by short pulses of completely saturating white light pulse (0.4–0.8 s, 1000–5000 μmol photons m^−2^ s^−1^), in order to record F_m_ (F_v_ = F_m_ − F_0_) (see [75] for reference).

#### 4.5.3. Photosynthetic Pigment Analysis

Chlorophyll *a*, chlorophyll *c*, and fucoxanthin (Fucox) were analyzed on the final day of the experiment. Gametophytes were carefully detached from each Petri dish, filtered through a 20 µm mesh, and placed in 2 mL Eppendorf tubes. Gametophytes (0.05 g FW) were incubated in 200 µL Dimethylsulfoxide (DMSO) for 20 min in the dark. This extract was centrifuged at 10,000 rpm for 5 min at 4 °C, until a supernatant and a precipitate of gametophytes were obtained. Then, the supernatant was placed in a spectrophotometer microplate to read absorbances. To the obtained precipitate (gametophytes), an additional 20 min incubation was performed using 700 µL 90% (*v*/*v*) acetone. The acetone extracts were then centrifuged at 10,000 rpm for 5 min at 4 °C. The concentrations of all pigments were calculated according to the methodology of Seely et al. [76] for DMSO and acetone (90%) extraction. Absorbance readings were obtained using a microplate spectrophotometer (Thermo Scientific, TECAN, Infinite 200Pro) with 400 µL of samples. Chl *a*, Chl *c*, and Fucox contents (mg g^−1^ WW) were calculated based on absorbance measurements from DMSO (1) at 480.0 nm (A480), 582.0 nm (A582), 631.0 nm (A631), and 665.0 nm (A665) and from 90% (*v*/*v*) acetone (2) at 470.0 nm (A470), 581.0 nm (A581), 631.0 nm (A631), and 664.0 nm (A664), using the following equations:

(1) DMSO extract:

Chl *a* (g L^−^^1^) = A_665_/72.8

Chl *c* (g L^−^^1^) = (A_631_ + A_582_ − 0.297A_665_)/61.8

Fucox (g L^−^^1^) = (A_480_ − 0.722 (A_631_ + A_582_ − 0.297A_665_) − 0.049A_665_)/130

(2) Acetone extract:

Chl *a* (g L^−^^1^) = A_664_/73.6

Chl *c* (g L^−^^1^) = (A_631_ + A_581_ − 0.3A_664_)/62.2

### 4.6. Data Analysis

The effects of the experimental factors (pH, light, and temperature) on the physiological responses of *M. pyrifera* gametophytes (Chlorophyll *a*, fluorescence, and photosynthetic pigment concentrations) were analyzed via two-way analysis of variance (ANOVA). To analyze the data obtained from the experiments involving the specific photosynthesis inhibitors, a three-way ANOVA was applied, considering the factors of pH, light/temperature, and the type of inhibitor. In all of the datasets, outliers were detected through the Bonferroni outlier test [77], using the “Bonferroni outlier Test” function of the “car” package in R. Then, verification of the assumptions of normality and homoscedasticity was carried out using the Shapiro–Wilk and Levene tests, respectively. In cases where the normality assumption was not fulfilled, a logit transformation was carried out. A post hoc Tukey test (*p* < 0.05) was applied when a significant effect of independent variables was observed. All statistical analyses were performed using the R v4.2.2. software (www.r-project.org) (accessed on 19 January 2023). 

## 5. Conclusions

The main conclusion of our study is that the Ci uptake mechanisms in gametophytes of the giant kelp *M. pyrifera* differ from those described in adult individuals. Specifically, gametophytes rely more on the CA_ext-_mediated pathway for using bicarbonate (HCO_3_^−^) to support photosynthesis, while adults primarily use an AE-type transporter for taking up HCO_3_^−^. Our results suggest that gametophytes exhibit high photosynthetic plasticity, able to adapt to varying environmental conditions such as light, temperature, and pH. While the lowered pH (increased CO_2_ availability) did not significantly enhance photosynthesis, it did increase the photosynthetic efficiency and quantum yield of gametophytes, suggesting potential benefits of OA for early life stages. Additionally, temperature and light conditions influenced the photosynthetic efficiency, with gametophytes showing the capacity to acclimate to different light intensities. These findings highlight the importance of environmental drivers in shaping the Ci utilization strategies and physiological responses of *M. pyrifera* gametophytes.

## Figures and Tables

**Table 1:** Two-way and three-way ANOVA results and significance values for effects of seawater pH_T_ treatments, light, and their interaction in Experiment 1: pH × light. Significant differences are indicated in bold.

Variable		Source of Variation	Df	Sum of Squares	Mean Square	*F*	*p*
CA specific inhibitors		pH_T_	1	136	136	0.819	0.37067
		Light	1	64	64	0.388	0.53696
		Inhibitor	2	23,318	11,659	70.289	**5.64 × 10^−14^**
		pH_T_ × Light	1	1472	1472	8.876	**0.00484**
		pH_T_ × Inhibitor	2	924	462	2.784	0.07348
		Light × Inhibitor	2	181	91	0.546	0.58328
		pH_T_ × Light × Inhibitor	2	10	5	0.030	0.97023
		Residual	41	6801	166		
		Total	52				
Direct HCO_3_^−^ uptake specific inhibitor		pH_T_	1	2626	2625.8	5.670	**0.0246**
		Light	1	10	10.3	0.022	0.8827
		Inhibitor	1	1590	1590.5	3.434	0.0748
		pH_T_ × Light	1	1824	1824.2	3.939	0.0574
		pH_T_ × Inhibitor	1	157	156.7	0.338	0.5656
		Light × Inhibitor	1	124	123.5	0.267	0.6097
		pH_T_ × Light × inhibitor	1	915	915.2	1.976	0.1712
		Residual	27	12,504	463.1		
		Total	34				
Chl *a*		pH_T_	1	0.00079	0.0007688	0.577	0.4583
		Light	1	0.0193442	0.0193442	14.5336	**0.001532**
		pH_T_ × Light	1	0.0039200	0.0039200	2.9452	0.105430
		Residual	16	0.0212960	0.00133		
		Total	19				
Chl *c*		pH_T_	1	0.046272	0.046272	76.6286	**1.692 × 10^−07^**
		Light	1	0.002599	0.002599	4.3044	0.0545066
		pH_T_ × Light	1	0.009946	0.009946	16.4706	**0.0009129**
		Residual	16	0.00962	0.000604		
		Total	19				
Fucoxanthin		pH_T_	1	0.0112813	0.012813	70.3539	**2.985 × 10^−07^**
		Light	1	0.0001012	0.0001012	0.6314	0.438459
		pH_T_ × Light	1	0.0025765	0.0025765	16.0677	**0.001014**
		Residual	16	0.0025656	0.0001603		
		Total	19				
Chl *c*/Chl *a* ratio		pH_T_	1	2.1125	2.1125	3.1467	0.0951083
		Light	1	13.5681	13.5681	20.2111	**0.0003668**
		pH_T_ × Light	1	0.0988	0.0988	0.1472	0.7062785
		Residual	16	10.7411	0.6713		
		Total	19				
Fucox/Chl *a* ratio		pH_T_	1	0.9622	0.9622	9.4816	**0.007186**
		Light	1	1.62530	1.62530	16.0155	**0.001028**
		pH_T_ × Light	1	0.06644	0.06644	0.6547	0.430320
		Residual	16	1.62372	0.10148		
		Total	19				
Alfa (*α*)		pH_T_	1	0.126995	0.126995	15.3919	**0.001213**
		Light	1	0.002045	0.002045	0.2479	0.625320
		pH_T_ × Light	1	0.000759	0.000759	0.0920	0.765564
		Residual	16	0.132012	0.008252		
		Total	19				
ETR_max_		pH_T_	1	851.4	851.4	1.0459	0.3216728
		Light	1	21,425.2	21,425.2	26.3208	**0.0001007**
		pH_T_ × Light	1	7714.7	7714.7	9.4774	**0.0071960**
		Residual	16	13,024.1	814.0		
		Total	19				
E_k_		pH_T_	1	410,680	410,680	6.3033	**0.023173**
		Light	1	589,571	589,571	9.0490	**0.008336**
		pH_T_ × Light	1	442,039	442,039	6.7846	**0.019156**
		Residual	16	1,042,450	65,153		
		Total	19				
F_v_/F_m_		pH_T_	1	0.083632	0.070899	72.1332	**4.09 × 10^−07^**
		Light	1	0.004149	0.004149	1.2811	0.275
		pH_T_ × Light	1	0.002299	0.002299	1.9732	0.181
		Residual	16	0.017394	0.001164		
		Total	19				

**Table 2:** Photosynthetic pigment concentrations estimated from *M. pyrifera* gametophytes incubated under different pH, light, and temperature conditions (Experiment 1: pH × light; Experiment 2: pH × temperature). Chlorophyll *a* (Chl *a*): (mg g^−1^ WW); Chlorophyll *c* (Chl *c*): (mg g^−1^ WW); Fucoxanthin (Fucox): (mg g^−1^ WW); Chlorophyll *c*/Chlorophyll *a* ratio (Chl *c*/Chl *a* ratio); Fucoxanthin/Chlorophyll *a* ratio (Fucox/Chl *c* ratio). Values represent the mean of 5 replicates ± SD.

**Experiment 1**	**pH**	**Light** **(µmol m^−2^s^−1^)**			**Pigment Concentration**		
			**Chl *a*** **(mg g^−1^ WW)**	**Chl *c*** **(mg g^−1^ WW)**	**Fucox** **(mg g^−1^ WW)**	**Chl *c*/Chl *a* Ratio**	**Fucox/Chl *a* Ratio**
	7.8	20	0.11 ± 0.03	0.15 ± 0.03	0.08 ± 0.01	1.45 ± 0.52	0.83 ± 0.32
		50	0.07 ± 0.03	0.22 ± 0.03	0.10 ± 0.01	3.24 ± 1.17	1.52 ± 0.52
	8.2	20	0.12 ± 0.07	0.10 ± 0.02	0.06 ± 0.02	0.94 ± 0.52	0.51 ± 0.11
		50	0.03 ± 0.01	0.08 ± 0.01	0.03 ± 0.01	2.45 ± 0.87	0.96 ± 0.15
**Experiment 2**	**pH**	**Temperature** **(°C)**					
	7.8	12	0.13 ± 0.03	0.12 ± 0.06	0.09 ± 0.01	0.88 ± 0.26	0.73 ± 0.16
		16	0.13 ± 0.03	0.09 ± 0.02	0.11 ± 0.01	0.69 ± 0.08	0.84 ± 0.12
	8.2	12	0.18 ± 0.05	0.10 ± 0.06	0.11 ± 0.02	0.55 ± 0.18	0.62 ± 0.11
		16	0.09 ± 0.01	0.06 ± 0.01	0.08 ± 0.01	0.69 ± 0.04	0.88 ± 0.07

**Table 3:** Photosynthetic parameters estimated from *M. pyrifera* gametophytes incubated under different pH, light, and temperature conditions (Experiment 1: pH × light; Experiment 2: pH × temperature). Photosynthetic efficiency (α): µmol m^−2^s^−1^. Maximal Electron Transport Rate (ETR_max_): µmol e^−1^m^−2^s^−1^. Irradiance for the initial saturation of ETR (E_k_): µmol m^−2^s^−1^. Maximal quantum yield (F_v_/F_m_): dimensionless. Values represent the mean of 5 replicates ± SD.

**Experiment 1**	**pH**	**Light** (**µmol m^−2^s^−1^)**	**Photosynthetic Parameter**			
			**α** **(µmol m^−2^s^−1^)**	**ETR_max_** **(µmol e^−1^m^−2^s^−1^)**	**E_k_** **(µmol m^−2^s^−1^)**	**F_v_/F_m_**
	7.8	20	0.32 ± 0.07	73.32 ± 29.62	240.14 ± 102.61	0.53 ± 0.03
		50	0.31 ± 0.14	47.14 ± 19.16	194.09 ± 150.14	0.53 ± 0.04
	8.2	20	0.17 ± 0.05	125.65 ± 44.12	824.07 ± 468.84	0.44 ± 0.05
		50	0.14 ± 0.08	20.91 ± 8.05	183.35 ± 87.90	0.38 ± 0.04
**Experiment 2**	**pH**	**Temperature** **(°C)**				
	7.8	12	0.13 ± 0.02	52.04 ± 8.62	399.98 ± 113.28	0.41 ± 0.01
		16	0.15 ± 0.03	67.64 ± 42.96	512.49 ± 463.86	0.33 ± 0.06
	8.2	12	0.29 ± 0.03	154.52 ± 89.34	558.14 ± 379.55	0.49 ± 0.01
		16	0.13 ± 0,02	76.95 ± 22.69	610.89 ± 219.26	0.29 ± 0.07

**Table 4:** Two- and three-way ANOVA results and significance values for effects of seawater pH_T_ treatments, temperature, and their interaction in Experiment 2: pH × temperature. Significant differences are indicated in bold.

Variable	Source of Variation	Df	Sum of Squares	Mean Squares	*F*	*p*
CA specific inhibitors	pH_T_	1	4813	4813	10.265	**0.00244**
	Temperature	1	208	208	0.444	0.50865
	Inhibitor	2	23,047	11,523	24.578	**4.95 × 10^−08^**
	pH_T_ × Temperature	1	328	328	0.701	0.40684
	pH_T_ × Inhibitor	2	1234	617	1.316	0.27801
	Temperature × Inhibitor	2	48	24	0.051	0.95046
	pH_T_ × Temperature × Inhibitor	2	898	449	0.957	0.39124
	Residual	47	22,036	469		
	Total	58				
Chl *a*	pH_T_	1	0.0001250	0.0001250	0.1144	0.739635
	Temperature	1	0.0090738	0.0090738	8.3012	**0.010857**
	pH_T_ × Temperature	1	0.0120050	0.0120050	10.9828	**0.004388**
	Residual	16	0.0174892	0.0010931		
	Total	19				
Chl *c*	pH_T_	1	0.0021013	0.0021013	1.0902	0.319
	Temperature	1	0.0056112	0.0056112	2.9114	0.1073
	pH_T_ × Temperature	1	0.000441	0.000441	0.2148	0.6492
	Residual	16	0.0308372	0.0019273		
	Total	19				
Fucoxanthin	pH_T_	1	0.0002048	0.0002048	1.1790	0.2936401
	Temperature	1	0.0001250	0.0001250	0.7196	0.4087771
	pH_T_ × Temperature	1	0.0030752	0.0030752	17.7041	**0.0006678**
	Residual	16	0.0027792	0.0001737		
	Total	19				
Direct HCO_3_^−^ uptake specific inhibitor	pH_T_	1	0.0093	0.0093	0.0216	0.884258
	Temperature	1	2.3219	2.3219	5.3748	**0.027682**
	Inhibitor	1	3.8610	3.8610	8.9378	**0.005643**
	pH_T_ × Temperature	1	0.8857	0.8857	2.0502	0.162876
	pH_T_ × Inhibitor	1	0.6803	0.6803	1.5747	0.219540
	Temperature × Inhibitor	1	0.5781	0.5781	1.3383	0.256780
	pH_T_ × Temperature × Inhibitor	1	0.0379	0.0379	0.0878	0.769070
	Residual	29	12.5277	0.4320		
	Total	36				
Chl *c*/Chl *a* ratio	pH_T_	1	0.13558	0.135584	4.9763	**0.04036**
	Temperature	1	0.00250	0.002503	0.0919	0.76573
	pH_T_ × Temperature	1	0.12890	0.128903	4.7311	**0.04496**
	Residual	16	0.43594	0.027246		
	Total	19				
Fucox/Chl *a* ratio	pH_T_	1	0.007987	0.007987	0.5588	0.465584
	Temperature	1	0.170134	0.170134	11.9038	**0.003292**
	pH_T_ × Temperature	1	0.029260	0.029260	2.0473	0.171721
	Residual	16	0.228680	0.014292		
	Total	19				
Alfa (*α*)	pH_T_	1	0.021258	0.021258	30.397	**4.721 × 10^−05^**
	Temperature	1	0.023899	0.023899	34.173	**2.483 × 10^−05^**
	pH_T_ × Temperature	1	0.038660	0.038660	55.280	**1.415 × 10^−06^**
	Residual	16	0.011189	0.000699		
	Total	19				
ETR_max_	pH_T_	1	15,619	1561.2	5.9981	**0.02622**
	Temperature	1	4801	4801.2	1.8438	**0.01934**
	pH_T_ × Temperature	1	10,853	10,853.5	4.1680	0.05804
	Residual	16	41,664	2604.0		
	Total	19				
E_k_	pH_T_	1	0.009677	0.0096772	1.6753	0.2139
	Temperature	1	0.000307	0.0003066	0.0531	0.8207
	pH_T_ × Temperature	1	0.002017	0.0020166	0.3491	0.5629
	Residual	16	0.092422	0.0057764		
	Total	19				
F_v_/F_m_	pH_T_	1	0.00337	0.00337	5.466	**0.03475**
	Temperature	1	0.11913	0.11913	193.074	**1.39 × 10^−09^**
	pH_T_ × Temperature	1	0.01393	0.01393	22.578	**0.00031**
	Residual	14	0.00864	0.00062		
	Total	17				

## Data Availability

Data is contained within the article.
